# The high-resolution NMR structure of the R21A Spc-SH3:P41 complex: Understanding the determinants of binding affinity by comparison with Abl-SH3

**DOI:** 10.1186/1472-6807-7-22

**Published:** 2007-04-02

**Authors:** Salvador Casares, Eiso AB, Henk Eshuis, Obdulio Lopez-Mayorga, Nico AJ van Nuland, Francisco Conejero-Lara

**Affiliations:** 1 Departamento de Química Física e Instituto de Biotecnología, Facultad de Ciencias, Universidad de Granada, Campus Fuentenueva s/n, 18071 Granada, Spain; 2 Bijvoet Center, Department of NMR Spectroscopy, Utrecht University, Padualaan 8, 3584CH Utrecht, The Netherlands

## Abstract

**Background:**

SH3 domains are small protein modules of 60–85 amino acids that bind to short *proline-rich *sequences with moderate-to-low affinity and specificity. Interactions with SH3 domains play a crucial role in regulation of many cellular processes (some are related to cancer and AIDS) and have thus been interesting targets in drug design. The decapeptide APSYSPPPPP (p41) binds with relatively high affinity to the SH3 domain of the Abl tyrosine kinase (Abl-SH3), while it has a 100 times lower affinity for the α-spectrin SH3 domain (Spc-SH3).

**Results:**

Here we present the high-resolution structure of the complex between the R21A mutant of Spc-SH3 and p41 derived from NMR data. Thermodynamic parameters of binding of p41 to both WT and R21A Spc-SH3 were measured by a combination of isothermal titration and differential scanning calorimetry. Mutation of arginine 21 to alanine in Spc-SH3 increases 3- to 4-fold the binding affinity for p41 due to elimination at the binding-site interface of the steric clash produced by the longer arginine side chain. Amide hydrogen-deuterium experiments on the free and p41-bound R21A Spc-SH3 domain indicate that binding elicits a strong reduction in the conformational flexibility of the domain. Despite the great differences in the thermodynamic magnitudes of binding, the structure of the R21A Spc-SH3:P41 complex is remarkably similar to that of the Abl-SH3:P41 complex, with only few differences in protein-ligand contacts at the specificity pocket. Using empirical methods for the prediction of binding energetics based on solvent-accessible surface area calculations, the differences in experimental energetics of binding between the two complexes could not be properly explained only on the basis of the structural differences observed between the complexes. We suggest that the experimental differences in binding energetics can be at least partially ascribed to the absence in the R21A Spc-SH3:P41 complex of several buried water molecules, which have been proposed previously to contribute largely to the highly negative enthalpy and entropy of binding in the Abl-SH3:P41 complex.

**Conclusion:**

Based on a deep structural and thermodynamic analysis of a low and high affinity complex of two different SH3 domains with the same ligand p41, we underline the importance of taking into account in any effective strategy of rational design of ligands, factors different from the direct protein-ligand interactions, such as the mediation of interactions by water molecules or the existence of cooperative conformational effects induced by binding.

## Background

Src-homology region-3 (SH3) domains are small protein modules mediating transient protein-protein interactions relative to a large number of cellular processes, many of them in connection with disease [1-4]. SH3 domains recognize proline-rich sequences through a highly-conserved mode of interaction. SH3-peptide interactions have been found to be highly promiscuous and relatively weak, with binding affinities ranging between 5 μM and 100 μM [5,6]. The binding site of SH3 domains consists of a hydrophobic surface with three shallow pockets defined by the side chains of preserved aromatic residues. These pockets are flanked by the n-src and RT loops, which play an important role in both the affinity and the specificity of binding [2,3,7]. A prominent feature of this type of interaction is the polyproline II (PPII) helical conformation for several residues of the peptide in the complexes, favouring the presence of proline residues in the sequences recognized by SH3 domains. There are however certain sequence positions in the ligand where proline is most frequently found, giving rise to consensus sequences for the SH3 ligands around a core sequence ΦPxΦP [2], being Φ a hydrophobic residue and x any residue. Two of the three hydrophobic pockets of the SH3 binding site are occupied by the side chains of each of the ΦP dipeptides of the core sequence. Additionally, the side chains of one each tryptophan and tyrosine residues donate a hydrogen bond to carbonyl groups of the peptide ligand. The third pocket, known as 'compass' or 'specificity' pocket, establishes additional interactions with less conserved residues of the ligand, conferring additional specificity to the binding and dictating the orientation of the ligand peptide chain along the binding site. This gives rise to class-1 or class-2 binding modes (see [8] for a comprehensive review of this type of interaction).

SH3 domains have become very important targets for drug design due to their involvement in a variety of diseases [4,9]. Notwithstanding the increasing amount of structural and functional information available about the recognition of proline-rich sequences by SH3 domains, the structural-thermodynamic determinants of binding affinity and specificity in these interactions are not fully understood. In particular, we do not understand well enough how changing a particular interaction at the binding interface modifies the binding Gibbs energy. This has limited the success in the design of high-affinity ligands for these small domains, even though some promising results have been obtained by rational design strategies [6] or by the use of libraries of N-substituted peptides [10].

One of the reasons for the relatively slow progress in understanding the determinants of SH3-peptide binding affinity and specificity is the difficulty in linking the structural information with a quantitative accounting of the energetics of these interactions [7,11-16]. In fact, the experimental thermodynamic magnitudes are often confusing. Isothermal titration calorimetry (ITC) studies with several SH3 domains and their ligands have always yielded strongly negative enthalpy changes of binding, partially compensated by negative entropy changes [11,15-18]. These results were unexpected as the binding interface is primarily hydrophobic. Several recent studies have proposed various possible contributions to the unusual thermodynamic features of this interaction: i.e., the reduction in the conformational motions of the protein [14], the folding of the peptide into the binding-competent PPII conformation [15] or the establishment of hydrogen-bond networks mediated by water molecules buried at the binding interface [16]. The relative importance of these effects in the global thermodynamic magnitudes of binding remains, however, obscure. An approach to discern between the contributions to the binding energetics coming from the ligand characteristics and those of the protein is to analyse the structural and thermodynamic determinants of binding of the same ligand to different SH3 domains.

Here we present the solution structure of the R21A mutant of the α-spectrin SH3 domain (Spc-SH3), both in its free form and bound to the decapeptide APSYSPPPPP (p41). We also show a complete thermodynamic characterization of the SH3-p41 interaction by calorimetric methods, together with an analysis of its effect upon the conformational dynamics of the SH3 domain using amide hydrogen-deuterium exchange (HX). The p41 peptide was initially designed as a high-affinity and high-specificity ligand for the Abl-SH3 domain [6] and the crystal structure of the complex was subsequently determined [12]. The Spc-SH3 domain also binds p41, although with a 100-times lower affinity [19]. The R21A mutation in the Spc-SH3 domain, near the tip of the RT-loop, produces a 3 to 4-fold increase in the affinity for p41, rendering this mutant more appropriate for thermodynamic analysis and structural resolution of the complex. The affinity enhancement is properly rationalized on the light of the three-dimensional structure of the complex. We also present a detailed comparison of the structures of the complexes between p41 and each of Abl-SH3 and R21A Spc-SH3, in conjunction with the thermodynamic magnitudes of binding, providing an interpretation of the differences observed on the basis of empirical methods for the prediction of binding energetics based on solvent-accessible surface area calculations. The results give insight into the determinants of binding of proline-rich peptides to these small domains and suggest an important role of water molecules buried at the binding interface in eliciting high affinity and specificity for these domains.

## Results

### The thermodynamics of binding of p41 to the R21A Spc-SH3 mutant domain

**Figure 1:**
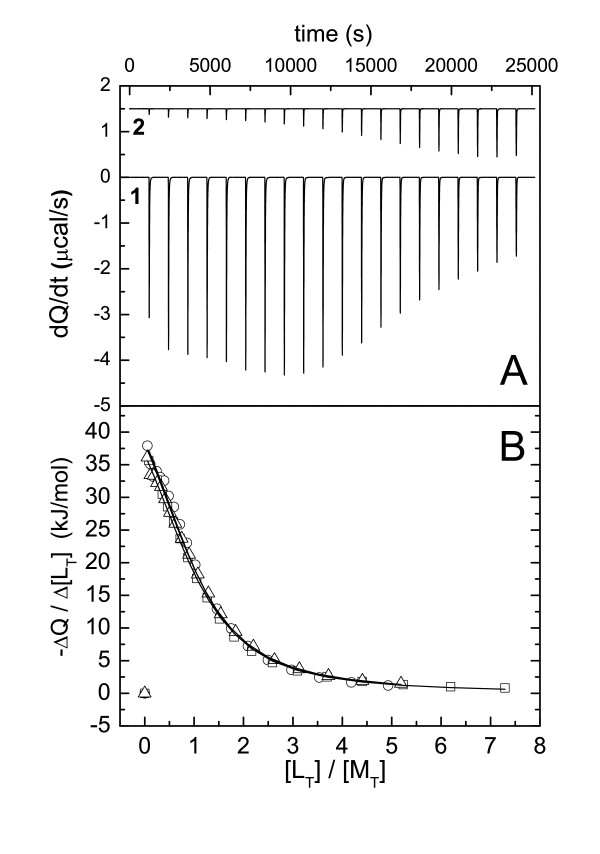
**Calorimetric titration of the R21A mutant of Spc-SH3 with the p41 peptide**. **A**. Experimental ITC thermograms obtained at pH 7.0 and 25°C by consecutive injections of variable volumes (ranging between 1.3 and 16 μl) of a p41 solution into the Spc-SH3 R21A mutant solution (1) and the buffer solution (2), respectively. **B**. Binding isotherms obtained at pH 7.0 using three different buffers (phosphate, squares; hepes, circles; and imidazole, triangles). Symbols represent the experimental data for the corrected and normalized heats produced by the binding after each injection. The continuous lines correspond to the best global fitting of the three isotherms using equation 1 (see Material and Methods).

The binding between the p41 decapeptide and the R21A Spc-SH3 mutant domain was studied by a combination of isothermal titration calorimetry (ITC) and differential scanning calorimetry (DSC) experiments. ITC experiments were performed at 25°C at both pH 3.0 and 7.0, using several buffers at a concentration of 20 mM. Similar ITC experiments were made with the WT Spc-SH3 domain for comparison. Figure 1A shows an example of a calorimetric titration of R21A Spc-SH3 with p41 at pH 7.0 and 25°C. The contribution of protonation/deprotonation processes to the binding enthalpy change, was estimated using three buffers differing in their ionization heats (Figure 1B). Table 1 summarizes the results of the ITC experiments.

Both Spc-SH3 variants bind the p41 ligand, although with low to moderate affinity. The affinities are much lower than that of the Abl-SH3 domain for p41 under similar conditions, which is in the range of 2 × 10^5 ^– 4 × 10^5 ^M^-1 ^[16]. In all cases the binding is driven by a large and favourable enthalpy change, partially compensated by an unfavourable entropy change. A similar thermodynamic signature is usually observed for the binding of proline-rich peptides to other SH3 domains [11,15-17]. The very small variation of the binding enthalpies with the ionization enthalpy of the buffers, indicates an insignificant proton exchange with the buffer upon binding, similarly to Abl-SH3 [16]. This is also supported by the small dependence of the binding enthalpy with pH for both protein variants.

At 25°C the affinity of the R21A Spc-SH3 mutant for p41 is about 3 to 4 times higher than that of the WT, and the magnitude of the enthalpy change increases significantly, indicating an increment in the number and/or strength of interactions between the domain and the ligand accompanying the replacement of the arginine side chain by alanine. Concomitantly, there is a decrease in the entropy change of binding, which results in a net Gibbs energy gain of about -3 kJ mol^-1^, at both pH values upon mutation. The increase in binding affinity cannot be related to any stability change induced by the R21A mutation, since both protein variants have almost identical stabilities as reported elsewhere [20].

**Figure 2:**
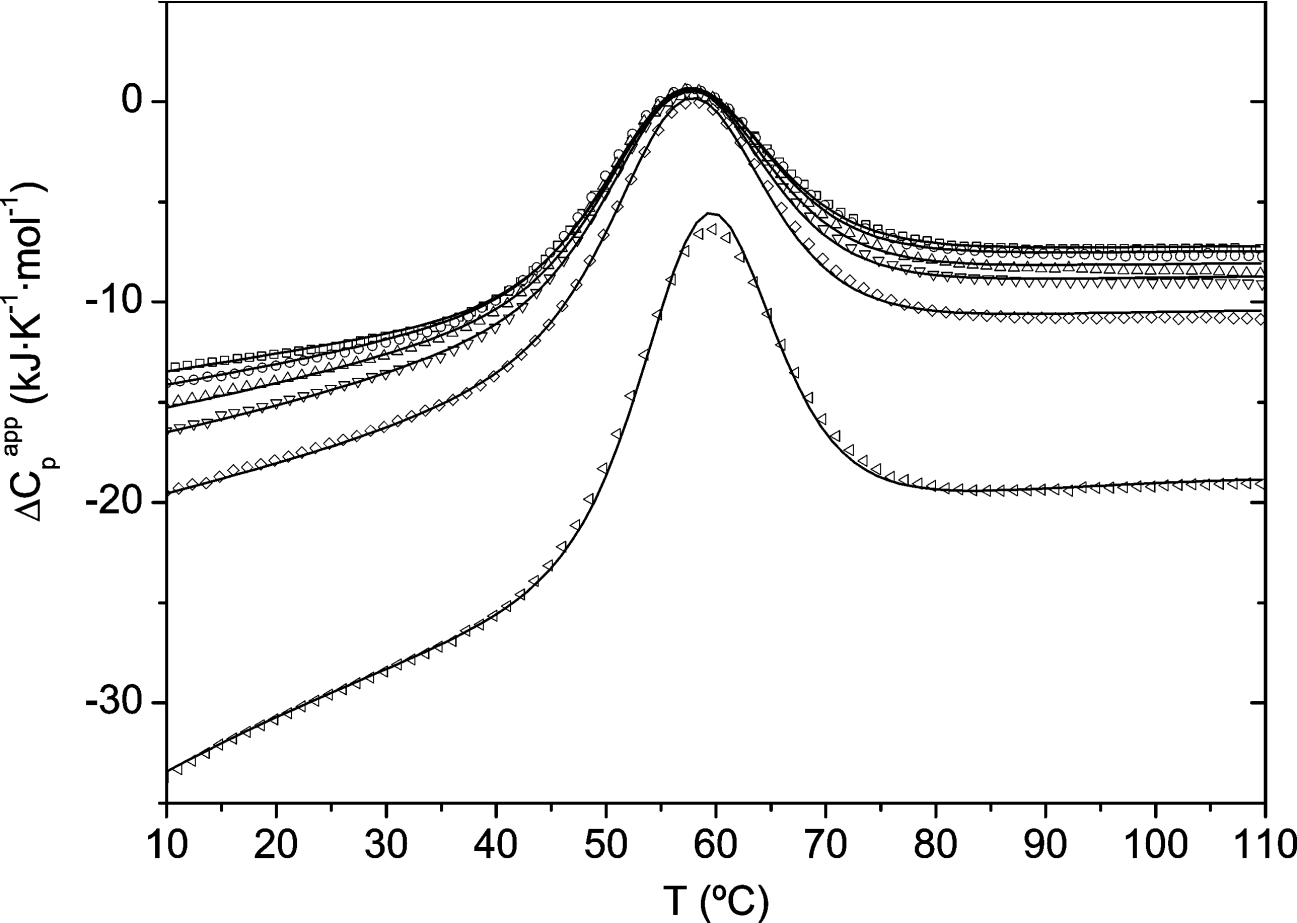
**Apparent molar heat capacity curves measured by DSC for the R21A Spc-SH3 domain in the presence p41**. The experiments were made in 20 mM glycine buffer, pH 3.0 at a protein concentration of 0.14 mM and variable concentrations of p41. Symbols represent the experimental curves for each p41/domain molar ratio (ranging from 0 to 9.8). The continuous lines correspond to the best global fitting using a model of coupled equilibria of binding and unfolding, as described in Supplementary material [Additional file 1].

Figure 2 shows the apparent molar heat capacity curves, ΔCpapp
 MathType@MTEF@5@5@+=feaafiart1ev1aaatCvAUfKttLearuWrP9MDH5MBPbIqV92AaeXatLxBI9gBaebbnrfifHhDYfgasaacH8akY=wiFfYdH8Gipec8Eeeu0xXdbba9frFj0=OqFfea0dXdd9vqai=hGuQ8kuc9pgc9s8qqaq=dirpe0xb9q8qiLsFr0=vr0=vr0dc8meaabaqaciaacaGaaeqabaqabeGadaaakeaacqqHuoarcqWGdbWqdaqhaaWcbaGaemiCaahabaGaemyyaeMaemiCaaNaemiCaahaaaaa@34D4@, measured by DSC at pH 3.0 in 20 mM glycine buffer, for different R21A/p41 molar ratios corresponding to saturation degrees between 0 and approximately 90%. The large decrease in ΔCpapp
 MathType@MTEF@5@5@+=feaafiart1ev1aaatCvAUfKttLearuWrP9MDH5MBPbIqV92AaeXatLxBI9gBaebbnrfifHhDYfgasaacH8akY=wiFfYdH8Gipec8Eeeu0xXdbba9frFj0=OqFfea0dXdd9vqai=hGuQ8kuc9pgc9s8qqaq=dirpe0xb9q8qiLsFr0=vr0=vr0dc8meaabaqaciaacaGaaeqabaqabeGadaaakeaacqqHuoarcqWGdbWqdaqhaaWcbaGaemiCaahabaGaemyyaeMaemiCaaNaemiCaahaaaaa@34D4@ with the p41 concentration is due to an increase in the amount of water excluded from the solution by the peptide. Another observation to notice is the increase in the apparent enthalpy of the unfolding transitions with the R21A/p41 molar ratio, consistently with the positive contribution from the enthalpy of dissociation of the complex to the unfolding process.

We have performed a global analysis of the ΔCpapp
 MathType@MTEF@5@5@+=feaafiart1ev1aaatCvAUfKttLearuWrP9MDH5MBPbIqV92AaeXatLxBI9gBaebbnrfifHhDYfgasaacH8akY=wiFfYdH8Gipec8Eeeu0xXdbba9frFj0=OqFfea0dXdd9vqai=hGuQ8kuc9pgc9s8qqaq=dirpe0xb9q8qiLsFr0=vr0=vr0dc8meaabaqaciaacaGaaeqabaqabeGadaaakeaacqqHuoarcqWGdbWqdaqhaaWcbaGaemiCaahabaGaemyyaeMaemiCaaNaemiCaahaaaaa@34D4@ curves using a simple model in which the protein/ligand binding and protein unfolding equilibria are coupled (Figure 2). This analysis is, with minor modifications, similar to that used previously for the analysis of the interaction between barnase and barstar [21]. The equations used to globally fit the ΔCpapp
 MathType@MTEF@5@5@+=feaafiart1ev1aaatCvAUfKttLearuWrP9MDH5MBPbIqV92AaeXatLxBI9gBaebbnrfifHhDYfgasaacH8akY=wiFfYdH8Gipec8Eeeu0xXdbba9frFj0=OqFfea0dXdd9vqai=hGuQ8kuc9pgc9s8qqaq=dirpe0xb9q8qiLsFr0=vr0=vr0dc8meaabaqaciaacaGaaeqabaqabeGadaaakeaacqqHuoarcqWGdbWqdaqhaaWcbaGaemiCaahabaGaemyyaeMaemiCaaNaemiCaahaaaaa@34D4@ data are described in detail in the Supplementary material [Additional file 1]. In this fitting procedure the equilibrium binding constant, *K*_*b*_, and the enthalpy change of binding, Δ*H*_*b*_, both at 25°C, have been fixed using the values measured by ITC under the same buffer conditions (Table 1). Additionally, the parameters of the thermal unfolding of the free R21A Spc-SH3 domain were determined independently and fixed in the global analysis. We also measured the partial molar *C*_*p *_curve of the free p41 peptide, which was fitted to a 4^th^-order polynomial and fixed in the global analysis. With all these restraints, the only adjustable parameter was the heat capacity change of binding, Δ*C*_*p*,*b*_, at 25°C. As shown in Figure 2 the quality of the fittings is very good, indicating the validity of this approach to estimate the heat capacity change of binding. This approach yielded a Δ*C*_*p*,*b *_value of -1.3 kJ K^-1 ^mol^-1 ^at pH 3.0 and 25°C, which is slightly larger in magnitude but in good agreement with the negative values reported by ITC for the complex between Abl-SH3 and p41 (between -0.7 and -1.0 kJ K^-1 ^mol^-1 ^at pH 7.0 and 25°C) [16]. Negative ΔC_p,b _values are characteristic of the burial of predominantly hydrophobic surface area upon ligand binding, a feature typical of the complexes between SH3 domains and their ligands.

### The structure of the free and bound form of R21A Spc-SH3

**Figure 3:**
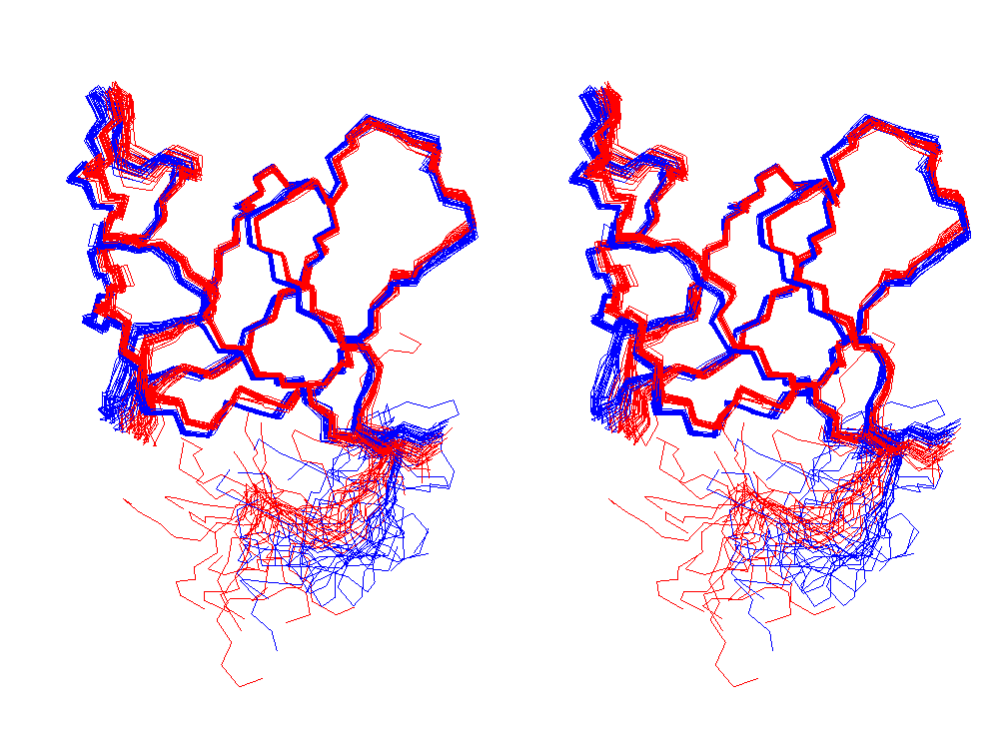
**Stereo view of the ensemble of 20 NMR structures of the free and bound form of R21A Spc-SH3**. The lowest-energy, water-refined structures of the free (blue) and bound (red) forms were superimposed on the backbone atoms (N,Ca,C') of residues 7–60.

**Figure 4:**
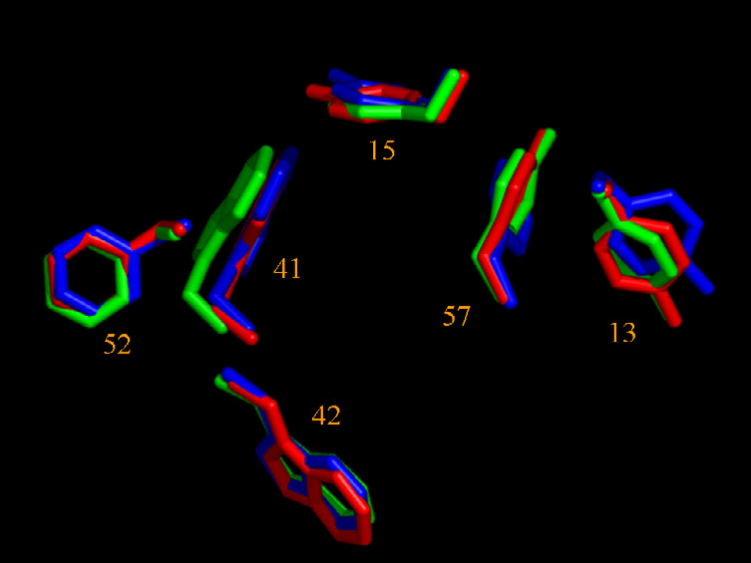
**Comparison between R21A Spc-SH3 with WT α-spectrin**. The lowest-energy free (green) and bound (red) NMR structures of R21A-SH3 were superimposed on the WT X-ray structure (1SHG, blue) for backbone atoms (N, Ca, C') of residues 7–60. Only the aromatic side chains are numbered and presented in sticks. Note that all aromatics, except Trp42, are important for binding.

Except for the labile side-chain hydrogen atoms, all proton resonances in the free as well as in the bound form were assigned from 2D TOCSY and 2D NOESY ^1^H-NMR spectra. These full assignments and the high quality of the 750 MHz spectra were crucial for the success of the automatic structure determination of both forms using a combined CANDID and ARIA protocol (see Material and Methods). Figure 3 shows a stereo representation of the superposition of the free on the bound R21A Spc-SH3 ensembles of structures. Both ensembles fulfil very well the experimental data and are of extremely high quality, as can be seen from the high percentage of the residues in the favoured region of the Ramachandran plot (Table 2). The structure of the free and bound forms are closely similar, with a pairwise RMSD of 0.64 Å and 1.35 Å for the backbone N,Ca,C' atoms and all heavy atoms of residues 7–60, respectively. They are both also very similar to the X-ray WT structure (pdb code: 1shg [22]) with a backbone pair wise RMSD of 0.71 Å and 0.49 Å for residues 7–60 of the free and the bound form, respectively. Figure 4 highlights the close similarity between the three structures, as the aromatic residues present in Spc-SH3 are coordinated in a very comparable way. All these residues except Trp42 are important for binding polyproline sequences. Moreover, the unfavorable torsion angles for residue 47 found in the WT X-ray structure are also present in all structures of both NMR ensembles.

### The structure of the R21A Spc-SH3:P41 complex

**Figure 5:**
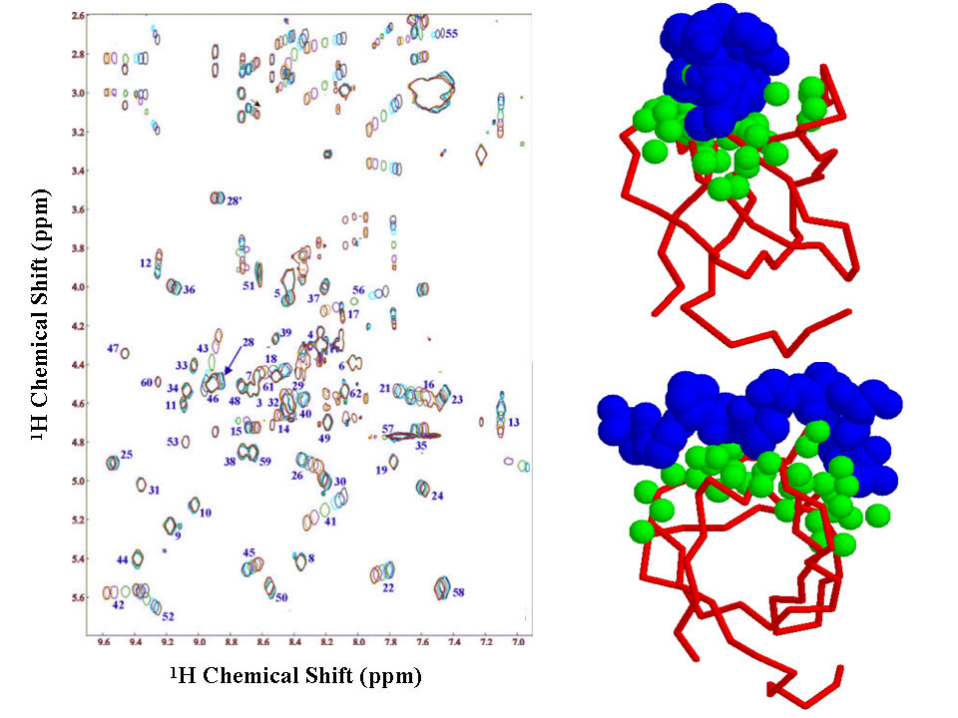
**Chemical shift perturbations resulting from the binding of p41 to R21A Spc-SH3**. Left panel: The *finger-print *region of the different TOCSY spectra recorded for the six titration points with p41. From black to dark blue the p41 concentration is increased. Only Cα H-NH cross-peaks are indicated. Right panel: Chemical shift perturbations for all protons >0.1 ppm (green balls) derived from the TOCSY experiments, mapped on a model of the complex of α-spectrin SH3 (red) with p41 (blue spheres) obtained from super-position on the Abl-SH3:P41 complex.

**Figure 6:**
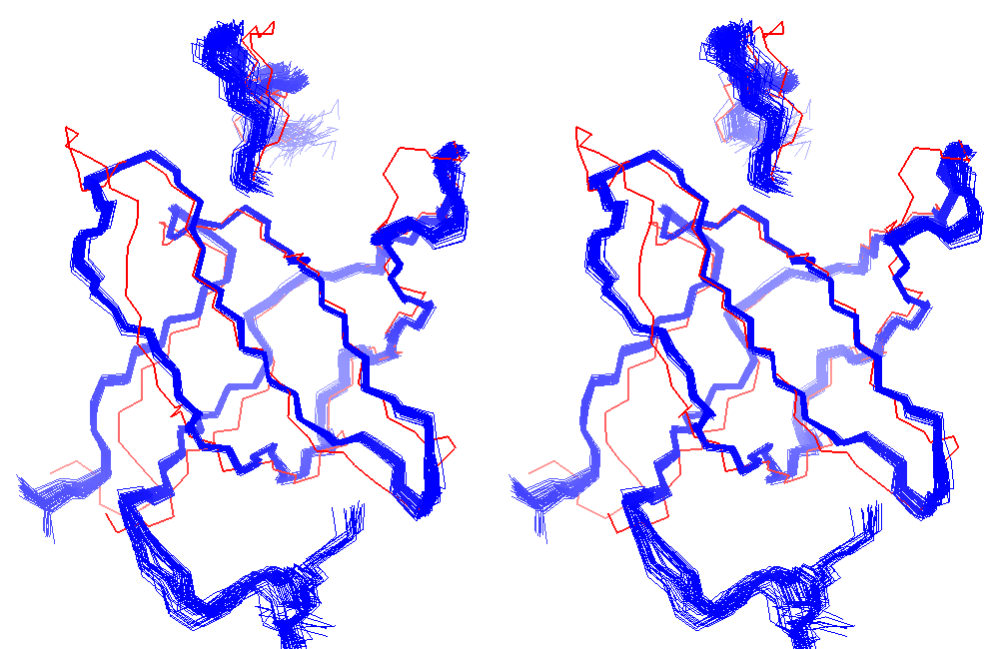
**Stereo view of the ensemble of 20 lowest interaction energy structures of the R21A Spc-SH3:P41 complex**. The structures (blue ribbons) are superimposed on the X-ray structure of the Abl-SH3:P41 complex (pdb 1BBZ, red) for comparison. Superposition was done on the backbone N,Ca,C' atoms of p41 and residues 8–17, 21–35, 39–45 and 50–59 of R21A Spc-SH3 to 2–11, 15–29, 34–40 and 45–54 of Abl-SH3, respectively.

The structure of the complex between R21A Spc-SH3 with the p41 peptide was obtained by a two-step docking protocol using the HADDOCK software [23]. Ambiguous interaction restraints were obtained by titrating a solution of R21A Spc-SH3 with p41 and subsequent recording of 2D-TOCSY spectra (Figure 5A). The most significant changes in the chemical shift of the TOCSY cross-peaks can be observed at the *RT loop *(residues 13–23 and 26), the beginning of the β-*III strand *(residues 40–43), the *3*_*10*_*-helix *(residues 55–57) and the F52 residue, as it was expected since they constitute the main structural elements involved in the binding to p41 in Abl-SH3. Figure 5B shows all protons (including backbone amide, Hα and side-chain protons) that shift more than 0.1 ppm in a model of the complex that was created by superimposing Spc-SH3 on the X-ray structure of the Abl-SH3:P41 complex (pdb 1bbz [12]). Ambiguous interaction restraints (AIRs) were defined between each of these protons and all atoms of the p41 ligand and used as input in the first HADDOCK run. An ensemble of 20 lowest interaction energy water-refined complex structures was then used to assign and identify NOEs within p41 and between the protein and ligand. The 33 AIRs and 38 intermolecular NOEs are collected in Table 1 of the Supplementary material [Additional file 1]. All AIRs, intra- and intermolecular NOEs were used as input in a second HADDOCK run (for details see Materials and Methods). The fifty lowest interaction energy structures were refined in shell of water. All 50 structures fall within 1 cluster using a cutt-off of 0.5 Å. The 20 water-refined lowest interaction energy structures were used for further analysis. The ensemble of these 20 NMR structures fulfils all experimental data; no violations greater than 0.3 Å of the NOE-derived or interaction restraints were found (Table 3). Figure 6 shows a stereo representation of the final R21A Spc-SH3:P41 ensemble of structures derived from the HADDOCK protocol superimposed on the X-ray structure of the Abl-SH3:P41 complex.

### The effect of p41 binding upon conformational flexibility of R21A Spc-SH3

**Figure 7:**
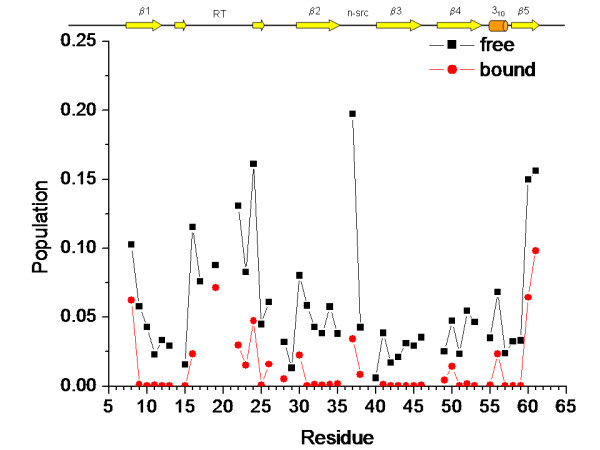
**The effect of p41 binding upon conformational flexibility in the R21A Spc-SH3 domain**. The population of conformational states of the protein having each residue in a disordered, solvent-exposed conformation was calculated from the experimental HX rate constants as described in Methods. Black squares and red circles correspond to the free and bound forms, respectively. A cartoon showing schematically the location of the secondary structure elements is placed on top of the graph.

HX rates, *k*_*hx*_, have been measured at pH* 3.0 and 27.1°C for the R21A Spc-SH3 domain, both in the free form and in the presence of the p41 peptide at a saturation degree of 96%. Reliable data (collected in Table 2 of the Supplementary material [Additional file 1]) could be extracted for 44 residues in the free form and 42 residues in the bound form of the possible 59. From these experimental data the population of states of the protein having each residue in a disordered, HX-competent conformation was calculated as described in Methods, for both the free and p41-bound forms of R21A Spc-SH3. It is evident from Figure 7 that the free domain has a considerable conformational flexibility, particularly high at the loops, the chain ends and the edges of secondary structure elements. The binding of p41 results in a strong stabilisation for many residues throughout the whole protein sequence, not only limited to those residues involved directly in the binding interface. Conformational flexibility is dramatically reduced at the central regions of the secondary structure elements of the domain, i.e., the five β-strands, the short 3_10 _helix, and a short stretch of β-sheet formed by residues 15 and 25 in the long RT-loop. Residues at more flexible regions in the free form also show great stabilising effects, in particular at the RT- and n-src loops, likely due to their involvement in p41 binding. Similar effects were observed for the binding of proline-rich peptides to other SH3 domains [14,24].

## Discussion

The combined use of CANDID, ARIA and HADDOCK has resulted in high quality structures of the R21A mutant of the α-spectrin SH3 domain when it is free in solution and bound to the proline-rich ligand p41. We found that CANDID was more efficient than ARIA in the automatic assignment of NOEs, while the resulting structures showed worse structural statistics. This prompted us to use CANDID for the automatic assignment and ARIA to calculate and refine the structures of R21A Spc-SH3 in the free and bound form. This approach resulted in high-quality structures for both forms (see Table 2) that are closely similar and highly comparable to the WT X-ray structure. The success of the approach can be attributed to the use of a single high-quality 750 MHz 2D NOESY spectrum and to the complete set of assignments of all proton frequencies in both cases.

### Comparison between the R21A mutant and WT Spc-SH3: the role of residue 21 in binding affinity

**Figure 8:**
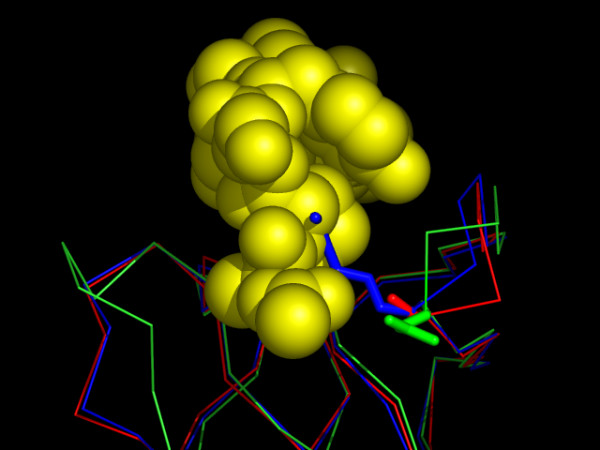
**The SH3:P41 interface**. The structure of the R21A Spc-SH3:P41 complex (backbone of SH3 in red, p41 colored yellow in space-filled representation) and the X-ray structure of Abl-SH3:P41 complex (only backbone of SH3 is shown, in green) has been superimposed on the backbone of WT Spc-SH3 (blue). Residue 21 in Spc-SH3 and the corresponding residue 15 in Abl-SH3 are represented by sticks. The long side chain at position 21 (Arg, blue sticks) in the WT structure crosses the binding interface of SH3 for p41 and explains the increase in binding affinity when replaced by an alanine (mutant R21A Spc-SH3, red sticks).

We have shown by the use of ITC, that the binding affinity of α-spectrin SH3 for p41 increases about 3- to 4-fold upon replacement of the arginine at position 21 for an alanine residue. The mutation, however, does not have any important effect upon the global or local stability of the domain as indicated by DSC and HX measurements [20]. Having both WT and mutant structures, we can now rationalize the difference in affinity for p41. The longer side chain at position 21 in the WT structure crosses the binding interface of SH3 (Figure 8), thus hindering tight binding of the p41 ligand to WT α-spectrin SH3. Although the corresponding residue in Abl-SH3 is comparable in side-chain length (Asn15), the local difference in the backbone structure of the RT loop of Abl-SH3 makes this side chain pointing away from the binding cleft, enabling tight binding in this case.

### Comparison between the R21A Spc-SH3:P41 and Abl-SH3:P41 complexes: rationalizing the differences in binding affinity

**Figure 9:**
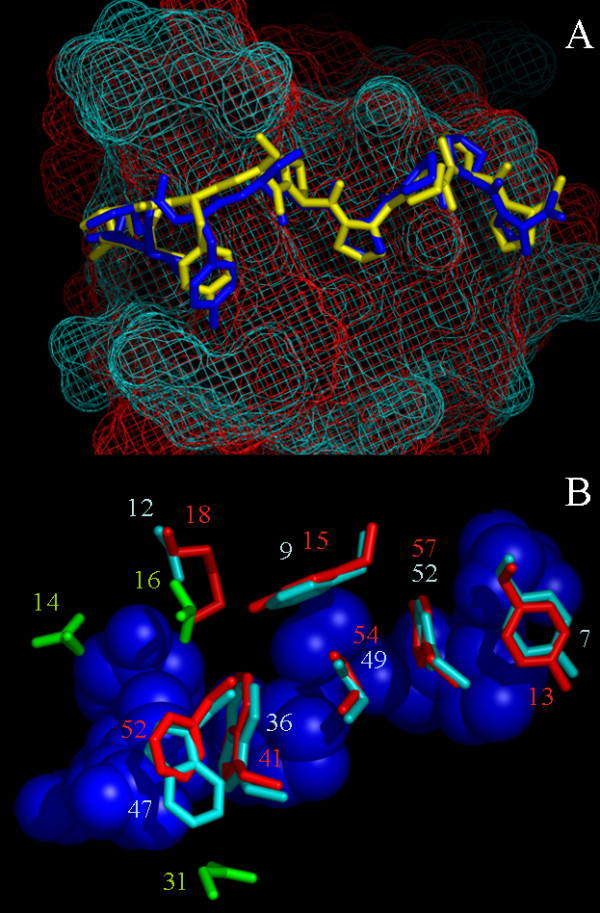
**The R21A Spc-SH3 and Abl-SH3 p41-binding interface**. **A**. Stick representation of p41 in complex with R21A Spc-SH3 (p41 yellow, SH3 red mesh) and with Abl SH3 (p41 blue, SH3 cyan mesh). Structural differences are mainly around Tyr4 due to the larger side chain of its H-bond partner in SH3 (Lys18 in Spc, Ser12 in Abl). **B**. Residues of SH3 involved in binding (R21A Spc-SH3 red, Abl SH3 cyan, p41 blue). Residues 14, 16 and 31 in Abl-SH3 (colored green) show contacts (LIGPLOT analysis [24]) in Abl-SH3 that are not present in Spc-SH3.

Figure 8 shows the binding interfaces of the R21A Spc-SH3:P41 complex obtained in this study and that of the Abl-SH3:P41 complex derived from X-ray crystallography. The p41 ligand adopts a very similar conformation in both complexes (Figure 9A). All structures in all iteration stages of the HADDOCK protocol showed unique orientation of the p41 peptide in agreement with a class 1 binding mode. Small structural differences are mainly around Tyr4, likely due to adapting to the larger side chain of its H-bond partner in Spc-SH3 (Lys18 in Spc, Ser12 in Abl). The aromatic residues and the Pro54 in Spc-SH3 (Pro49 in Abl), highly preserved among SH3 domains and critical for binding polyproline motifs, are positioned in the same way in both complexes as can been seen in Figure 9B. We used LIGPLOT [25] to analyze hydrophobic contacts and hydrogen bonds within the R21A Spc-SH3:P41 and Abl-SH3:P41 complexes and the results are summarized in Table 4. Generally the same contacts are found in the two protein-ligand complexes, although a few differences are observed. More specifically, residues 14, 16 and 31 in Abl-SH3 (presented as green sticks in Figure 9B) show contacts with p41 that are not identified in the R21A Spc-SH3:P41 complex. This could in principle contribute, at least partially, to the higher binding enthalpy and affinity of the Abl-SH3 domain for p41 relative to that of the Spc-SH3 domain (see Table 1).

The interaction between R21A Spc-SH3 and p41 buries 686 ± 11 Å^2 ^of non-polar area and 241 ± 11 Å^2 ^of polar area, very similar to the values of the Abl-SH3:P41 complex (708 ± 5 Å^2 ^and 282 ± 15 Å^2^, respectively), in good agreement with the structural details of both complexes.

One of the simplest and most frequently used methods to derive thermodynamic parameters of binding from experimental structural information is based upon applying semi empirical coefficients to the changes in polar and apolar solvent-accessible surface areas (ASA) during binding [26-29]. We have used the method of Baker and Murphy [29] assuming a rigid-body binding model (see Material and Methods) and ignoring initially the possible participation of buried water molecules in the complexes. With these assumptions, the predictions for the binding heat capacity changes (around -1 kJ·K^-1^·mol^-1^, Table 5) are in reasonably good agreement with the experimental values (see above). Conversely, the predicted binding enthalpies and entropies are completely flawed: both the calculated enthalpic and entropic terms of the binding energy are positive, consistent with a predominantly hydrophobic interaction. More importantly, the predicted differences in the thermodynamic magnitudes between both complexes do not reflect the large experimental differences in binding enthalpy, entropy and Gibbs energy, which, therefore, cannot be justified on the basis of only the SH3:P41 interaction.

Several important contributions to the binding energetics have been neglected in these calculations, such as the folding of the p41 peptide [15], the change in the conformational dynamics of the protein induced by ligand binding [14,15], and the role of long-lived, bound water molecules in mediating binding interactions [16,30-33]. The first of these contributions should not have an influence here since the two complexes share the same ligand in a very similar bound conformation.

On the other hand, the results of the HX analysis show that binding of p41 strongly reduces conformational flexibility of the R21A Spc-SH3 domain. It is thus plausible that this effect has an important impact on the thermodynamic magnitudes of binding, as it has been discussed elsewhere [14,20]. In fact, both of the enthalpies and the entropies of the Spc-SH3 conformational fluctuations under native conditions have been found elsewhere to be large, positive and mutually compensating [34]. Therefore, a decrease in the probability of occurrence of these fluctuations on p41 binding may well account for a significant portion of the negative enthalpy and entropy of binding as observed experimentally. It is also possible that the reductions in conformational dynamics of Spc-SH3 and Abl-SH3 on p41 binding are dissimilar enough to account for part of the observed differences in binding thermodynamics. To confirm such possible contributions would imply, however, an extensive comparative study of the effect of ligand binding to not only Spc-SH3 but to Abl-SH3 as well, which will be the scope of future work.

**Figure 10:**
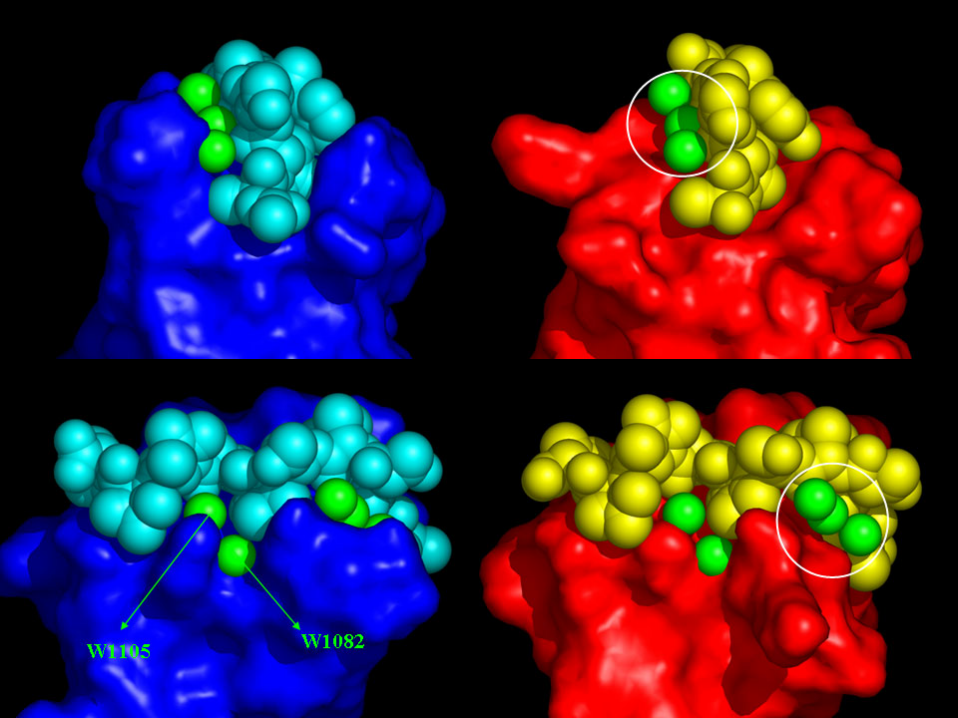
**Involvement of water in the SH3:P41 binding interface**. The five water molecules (green spheres) that were proposed to contribute to the highly favorable binding enthalpy in the Abl-SH3:P41 complex (left panel) were modeled into the R21A Spc-SH3:P41 structure (right panel) by direct superposition of the two complexes. Waters W1082 and W1105 (labeled in the Abl-SH3:P41 complex) are similarly buried in the two complexes, while the remaining three waters (highlighted by a white circle) are exposed in the R21A Spc-SH3:P41 complex and likely not anymore present in the binding interface.

Recent calorimetric studies have proposed that five water molecules, found buried into the binding interface in the crystal structure of the Abl-SH3:P41 complex, may be responsible for the highly favorable binding enthalpy, partially compensated by a large binding entropy [16]. We modeled these water molecules into the R21A Spc-SH3:P41 structure by direct superposition of the two complexes (Figure 10). Two of these five waters (W1082 and W1105) appear similarly buried in the two complex interfaces, while the other three seem no longer buried in the R21A Spc-SH3:P41 complex, suggesting that they are likely not mediating this interaction. To have a rough estimate of the differential energetic impact of these buried water molecules upon the two complexes, we have recalculated the changes in ASA on binding considering the five buried water molecules as part of the Abl-SH3:P41 complex and only two buried water molecules as part of the R21A Spc-SH3:P41 complex (Table 5). The predicted differences in binding energetics between Abl-SH3 and Spc-SH3 are now in a better qualitative agreement with the experimental ones providing a plausible explanation for the much higher binding affinity of Abl-SH3 for p41. This supports an important role for buried water molecules in mediating the SH3-peptide interactions, which are specially optimized in the Abl-SH3 domain resulting in high binding affinity.

## Conclusion

The combined use of modern automatic structure calculation and docking methods have resulted in high quality structures of the R21A mutant of the α-spectrin SH3 domain in the absence and presence of proline-rich ligand p41. The high resemblance between the R21A Spc-SH3 domain structure in its free and in its p41-bound form implies that the binding interface is completely predetermined and no major conformational changes are needed to adapt to the ligand structure upon binding. Based on a deep structural and thermodynamic analysis of the R1A Spc-SH3 in complex with p41 in comparison with the complex between another SH3 domain, Abl-SH3, with higher affinity to the same ligand p41, we underline the importance of taking into account in any effective strategy of rational design of ligands, factors different from the direct protein-ligand interactions, such as the mediation of interactions by water molecules or the existence of cooperative conformational effects induced by binding.

## Methods

### Sample preparation

The WT and R21A mutant SH3 domains were over-expressed in *E. coli *cells and purified as described previously [35]. The DNA encoding the R21A mutant Spc-SH3 domain was obtained by standard PCR techniques [36]. Sample concentration was determined spectrophotometrically using extinction coefficients of 16147 M^-1 ^cm^-1 ^at pH 7.0 and 15513 M^-1 ^cm^-1 ^at pH 3.0, determined as described elsewhere [37].

The synthetic decapeptide p41 (APSYSPPPPP, both acetylated and methylated in its termini) was purchased to Diverdrugs S.L. (Barcelona, Spain). Stock solutions of p41 were prepared by dissolving the lyophilized peptide in the buffer, filtering the solution to remove any insoluble material and then readjusting the pH. The peptide concentration was determined by UV absorption at 276 nm, using an extinction coefficient of 1450 M^-1 ^cm^-1^, as corresponding to its single tyrosine residue.

### Isothermal titration calorimetry

ITC experiments were performed at 25°C in a high-precision MCS titration calorimeter (Microcal Inc., Northampton, MA). Prior to the experiments the protein was extensively dialyzed at 4°C against the appropriate buffer. The solutions of p41 peptide were prepared by dissolving the lyophilized material directly into the buffer and subsequently adjusting the pH of the solution. All buffers and solutions were filtered and degassed and equilibrated to the working temperature prior to each experiment. Typically, a ≈ 0.1 mM protein solution in the calorimeter's cell was titrated with the ligand solution (at a concentration about 4 – 5 mM). Due to the relatively low binding affinities, titrations were made by using a profile of injection volumes of the ligand solution in order to define better the titration curve. An independent identical titration with buffer in the cell was made with the same ligand solution to determine the corresponding heats of dilution. The dilution thermogram was subtracted from that obtained for the titration of the protein. The area under each peaks of the net thermogram was integrated to determine the heat produced by the binding between the ligand and the protein after each injection. To account for the contribution of protonation/deprotonation processes to the binding enthalpy, the experiments were repeated at each pH using three buffers differing in their ionization heats. The binding constant, *K*_*b*_, and the enthalpy change of binding, Δ*H*_*b*_, were determined by global fitting of the three binding isotherms measured in different buffers, using a model of binding to a single set of identical sites, according to the equation:

ΔQΔ[L]T=VC⋅(ΔHb+p⋅ΔHi)2[1−1+Kb[L]T−nKb[M]T(1+Kb[L]T−nKb[M]T)2−4nKb2[M]T[L]T]
 MathType@MTEF@5@5@+=feaafiart1ev1aaatCvAUfKttLearuWrP9MDH5MBPbIqV92AaeXatLxBI9gBaebbnrfifHhDYfgasaacH8akY=wiFfYdH8Gipec8Eeeu0xXdbba9frFj0=OqFfea0dXdd9vqai=hGuQ8kuc9pgc9s8qqaq=dirpe0xb9q8qiLsFr0=vr0=vr0dc8meaabaqaciaacaGaaeqabaqabeGadaaakeaadaWcaaqaaiabfs5aejabdgfarbqaaiabfs5aejabcUfaBjabdYeamjabc2faDnaaBaaaleaacqWGubavaeqaaaaakiabg2da9maalaaabaGaemOvay1aaSbaaSqaaiabdoeadbqabaGccqGHflY1cqGGOaakcqqHuoarcqWGibasdaWgaaWcbaGaemOyaigabeaakiabgUcaRiabdchaWjabgwSixlabfs5aejabdIeainaaBaaaleaacqWGPbqAaeqaaOGaeiykaKcabaGaeGOmaidaamaadmaabaGaeGymaeJaeyOeI0YaaSaaaeaacqaIXaqmcqGHRaWkcqWGlbWsdaWgaaWcbaGaemOyaigabeaakiabcUfaBjabdYeamjabc2faDnaaBaaaleaacqWGubavaeqaaOGaeyOeI0IaemOBa4Maem4saS0aaSbaaSqaaiabdkgaIbqabaGccqGGBbWwcqWGnbqtcqGGDbqxdaWgaaWcbaGaemivaqfabeaaaOqaamaakaaabaGaeiikaGIaeGymaeJaey4kaSIaem4saS0aaSbaaSqaaiabdkgaIbqabaGccqGGBbWwcqWGmbatcqGGDbqxdaWgaaWcbaGaemivaqfabeaakiabgkHiTiabd6gaUjabdUealnaaBaaaleaacqWGIbGyaeqaaOGaei4waSLaemyta0Kaeiyxa01aaSbaaSqaaiabdsfaubqabaGccqGGPaqkdaahaaWcbeqaaiabikdaYaaakiabgkHiTiabisda0iabd6gaUjabdUealnaaDaaaleaacqWGIbGyaeaacqaIYaGmaaGccqGGBbWwcqWGnbqtcqGGDbqxdaWgaaWcbaGaemivaqfabeaakiabcUfaBjabdYeamjabc2faDnaaBaaaleaacqWGubavaeqaaaqabaaaaaGccaGLBbGaayzxaaaaaa@898D@

where ΔQΔ[L]T
 MathType@MTEF@5@5@+=feaafiart1ev1aaatCvAUfKttLearuWrP9MDH5MBPbIqV92AaeXatLxBI9gBaebbnrfifHhDYfgasaacH8akY=wiFfYdH8Gipec8Eeeu0xXdbba9frFj0=OqFfea0dXdd9vqai=hGuQ8kuc9pgc9s8qqaq=dirpe0xb9q8qiLsFr0=vr0=vr0dc8meaabaqaciaacaGaaeqabaqabeGadaaakeaadaWccaqaaiabfs5aejabdgfarbqaaiabfs5aejabcUfaBjabdYeamjabc2faDnaaBaaaleaacqWGubavaeqaaaaaaaa@35B3@ is the heat produced per mole of increment in the ligand concentration, V_C _is the active cell volume, [M]_T _and [L]_T _are the total concentrations of protein and ligand, respectively, *p *is the number of protons exchanged with the buffer per mole of ligand bound and ΔH_i _is the ionization enthalpy of the buffer.

### Differential scanning calorimetry

DSC experiments with the R21A mutant of Spc-SH3 in the presence of variable concentrations of p41 ligand were made using a VP-DSC microcalorimeter (Microcal Inc., Northampton, MA). A stock protein sample was extensively dialyzed at 4°C against the appropriate buffer and subsequently filtered and degassed. Samples for DSC were prepared by adding variable amounts of a stock solution of p41 peptide to the dialyzed protein sample. The protein concentration was fixed to 1 mg ml^-1 ^(0.14 mM), whereas the ligand concentration was varied between 0 and 1.42 mM. Apparent heat capacity curves, ΔCpapp
 MathType@MTEF@5@5@+=feaafiart1ev1aaatCvAUfKttLearuWrP9MDH5MBPbIqV92AaeXatLxBI9gBaebbnrfifHhDYfgasaacH8akY=wiFfYdH8Gipec8Eeeu0xXdbba9frFj0=OqFfea0dXdd9vqai=hGuQ8kuc9pgc9s8qqaq=dirpe0xb9q8qiLsFr0=vr0=vr0dc8meaabaqaciaacaGaaeqabaqabeGadaaakeaacqqHuoarcqWGdbWqdaqhaaWcbaGaemiCaahabaGaemyyaeMaemiCaaNaemiCaahaaaaa@34D4@, were obtained by subtraction of the instrumental baseline from the DSC thermogram obtained with the samples. In all experiments the temperature scan rate was 1°C min^-1^.

### NMR spectroscopy

Two-dimensional (2D) homonuclear ^1^H total correlation spectroscopy (TOCSY) and nuclear Overhauser enhancement spectroscopy (NOESY) spectra were recorded on a Bruker DRX 750 MHz spectrometer at 300 K. Firstly, these spectra were recorded on a 2 mM R21A-SH3 sample in 500 μl 90% H_2_O/10% D_2_O, 20 mM d5-glycine at pH 3.5. Then, p41 was added in 6 steps till a final concentration of 5.2 mM. After every addition, a 2D TOCSY spectrum was recorded, and after the final step also a 2D NOESY was recorded for the structure determination of the complex. Mixing times were 150 ms in the NOESY experiments and 76 ms in the TOCSY experiments. Spectra were processed with NMRPipe [38] and analysed using NMRView [39]. The ^1^H chemical shifts of the free form were assigned using standard methods [40] with the use of the WT Spc-SH3 assignments [41]. The ^1^H chemical shifts of the bound form were assigned by following the shift changes upon gradual titration of p41 into the SH3 sample by recording of 2D TOCSY spectra.

The chemical shift assignments for free and bound R21A Spc-SH3 were deposited into the BioMagResBank under accession codes 7305 and 7306, respectively.

### Structure calculations of the free and bound forms of R21A Spc-SH3

Distance restraints were derived from the 750 MHz 2D homonuclear ^1^H-^1^H NOESY spectra. After automatic peak picking, the diagonal, noise and artefact peaks (and peaks arising from p41 in the case of bound SH3) were removed manually. The remaining peaks were then used as input for automatic NOE assignment using the program CANDID [42,43]. The tolerance was set to 0.008 ppm in the direct dimension and to 0.015 ppm in the indirect dimension. The quality of the structures was improved in an iterative procedure where CANDID runs were followed by manual analysis to find additional resonance assignments using the NOE spectra and the preliminary structures as well as to improve the quality of the peak lists. Hydrogen-bond restraints derived from hydrogen-deuterium experiments [34] were defined if they were consistent with the secondary shift data, expected NOE contacts and the structures. Manual NOE peak assignments were generally not fixed in the CANDID runs but used to create accurate chemical shift lists, to check the consistency of subsequent CANDID runs and to check the manual assignments. The unambiguous and ambiguous assigned peaks in conjunction with H-bond restraints were subsequently used as input for final calculations with CNS [44] using the ARIA setup and protocols [45] that included correction of the distance bounds for spin diffusion effects. A total of 100 structures were finally refined in explicit water [46]. The twenty lowest-energy structures were further used for structural analysis (see Table 2). Validation of the structural quality was done using PROCHECK [47,48].

### Structure calculations of the R21A Spc-SH3:P41 complex

The structure of the complex between R21A Spc-SH3 and p41 was obtained in two steps using the HADDOCK software [24]. Thirty-three ambiguous interaction restraints (AIR) were defined for protons of SH3 with chemical shift perturbations bigger than 0.1 ppm to all atoms of the p41 ligand to 6Å (see table 1 in the Supplementary material [Additional file 1]). During the first step these AIRs were used together with the lowest energy R21A Spc-SH3 structure in the bound form and the X-ray structure of the p41 ligand in the Abl-SH3:P41 complex (pdb 1BBZ [12]) as input in HADDOCK. The protein was kept rigid during docking, the ligand semi flexible. During water refinement all unambiguous intra residue NOEs for the protein were included. Twenty out of 100 structures with the lowest interaction energies were selected for assigning NOEs within p41 and between the SH3 domain and the p41 ligand (see Table 1 in the Supplementary material [Additional file 1]). In the second step, all AIRs and all intra- and inter-molecular NOE-derived restraints were used during all docking steps. Docking was now started from the whole ensemble of 20 lowest-energy SH3 structures in the bound form and the X-ray structure of the ligand. Again the protein was kept rigid during docking, but now the ligand was left fully flexible. The final step of the structure refinement was done in explicit water. The twenty structures (out of 50 water-refined structures) with the lowest interaction energies were selected for further analysis (see Table 2).

The atomic coordinates of the free and bound forms of R21A Spc-SH3 and of the R21A Spc-SH3:P41 complex have been deposited in the Protein Data Bank (PDB accession code 2JM8, 2JM9 and 2JMA, respectively).

### Amide hydrogen-deuterium exchange

Amide hydrogen-deuterium exchange (HX) rates were measured by two-dimensional NMR for the free and p41-bound forms of the R21A Spc-SH3 domain, in 20 mM d_5_-glycine buffer, at pH* 3.0 (direct pH-meter reading) and 27.1°C, as described previously [20]. Samples were prepared by dissolving the salt-free lyophilized protein directly in the buffer, either in the presence or in the absence of the p41 peptide, and subsequently readjusting their pH* with DCl and NaOD solutions. Then, the samples were immediately transferred into the NMR probe for acquisition of a series of two-dimensional COSY spectra on a Bruker AMX-500 spectrometer. The final protein concentration was about 4.5 mM. The experimental HX rate constants, *k*_*hx*_, were calculated for each residue by fitting the intensity decay of the H_N_-H_α _cross peak to a single exponential decay function. Intrinsic HX rate constants for each amide proton, *k*_*int*_, were calculated as described elsewhere [49]. An EX2 mechanism was assumed for the HX of all residues under our experimental conditions as discussed previously [50]. The equilibrium constant, *K*_*hx*_, for the unfolding process that renders the amide hydrogen of any particular residue susceptible to exchange, was calculated as being *K*_*hx *_= *k*_*hx*_*/k*_*int*_. Finally, the population of conformational states of the protein having each residue in the unfolded, HX-competent conformation is obtained as *P*_*hx *_= *K*_*hx*_*/(1+K*_*hx*_*)*.

### Structure-based thermodynamic calculations

The thermodynamic magnitudes of p41 binding to Abl- and R21A Spc-SH3 domains were estimated from the changes in solvent-accessible surface areas (ΔASA) during molecular association as described elsewhere [29]. The calculations were assuming that the peptide and the protein have the same equilibrium conformations (backbone and side chains) in both the bound and unbound states, given by the experimentally-determined coordinates of the complexes, i.e., the ensemble of the 20 lowest-energy structures calculates here for the R21A Spc-SH3:P41 complex and the four models in X-ray crystal structure of the Abl-SH3:P41 complex (PDB code 1bbz).

The ASA values of the free and bound states were calculated using the program NACCESS [51] based upon the Lee & Richards algorithm [52]. Default parameters were used for the water probe radius (1.4 Å), z-slice (0.05 Å) and van der Waals radii.

To account for the effects of buried water molecules at the binding interface on the thermodynamic parameters we considered the presence of 5 water molecules buried in the Abl-SH3:P41 complex [16] and 2 water molecules buried in the R21A Spc-SH3:P41 complex (W1082 and W1105, Fig 9), whereas no buried water remains in the unbound state.

The ΔASA values were calculated with interface water molecules in the bound structures as if an extension of the protein. Water molecules present in the bound state were considered to contribute to the ASA of the unbound state with the ASA of a fully exposed water molecule (98.5 Å^2^). The water contribution to the binding entropy was estimated as the entropic cost of removing a water molecule from the bulk solvent and immobilizing it in the complex (about -30 J·K^-1^·mol^-1 ^[53]).

## Abbreviations

SH3, Src-homology domain 3; Spc-SH3, SH3 domain of chicken brain α-spectrin; Abl-SH3, SH3 domain of the Abl tyrosine kinase; p41, APSYSPPPPP decapeptide, both acetylated and methylated in its termini; AIR, Ambiguous Interaction Restraint; NMR, Nuclear Magnetic Resonance; NOESY, nuclear Overhauser enhancement spectroscopy; TOCSY, total correlation spectroscopy; DSC, differential scanning calorimetry; ITC, isothermal titration calorimetry; HX, amide hydrogen-deuterium exchange; pH*, pH-meter reading for deuterium oxide solutions uncorrected for isotopic effects.

## Authors' contributions

S.C. produced and purified the protein, assigned the NMR resonances and performed the calorimetry and HX studies. E.A. carried out most of the structure calculations and supervised H.E.. H.E. started the initial structural work. O.L-M. participated in the design of the study and supervised the work of S.C.. N.A.J.v.N. carried out the HADDOCK calculations and supervised the NMR work of S.C. and the structural work of E.A. and H.E.. F.C-L. participated in the design of the study, analysed the thermodynamic and HX data and supervised the work of S.C.. N.A.J.v.N. and F.C-L. wrote the manuscript, except for some parts of the Methods that were written by S.C. and E.A. All authors read and approved the final manuscript.

## Supplementary Material

Additional file 1 Thermodynamic analysis of protein-ligand binding using differential scanning calorimetry and two tables: Table 1: Ambiguous interaction restraints (AIR) and intermolecular NOE-derived distance restraints. Table 2: Apparent amide hydrogen-deuterium exchange rate constants and apparent Gibbs energies for the R21A Spc-SH3 domain at pH* 3.0 and 27.1°C, in its free form and in the presence of a 96% saturating concentration of the p41 peptide. description and equations used for analysis of DSC thermograms and list of AIR and SH3:P41 intermolecular NOEs and a list of amide hydrogen-deuterium exchange rate constants and apparent Gibbs energies. Click here for file

## Figures and Tables

**Table 1:** Thermodynamic parameters of the binding between p41 and the WT and R21A variants of the Spc-SH3 domain at 25°C measured by ITC^1^

**pH**	**Protein**	**K_b _(×10^-3 ^M^-1^)**	**ΔH_b _(kJ mol^-1^)**	**p**	**TΔS_b _(kJ mol^-1^)**	**ΔG_b _(kJ mol^-1^)**
7.0	R21A	18.1 ± 1.9	-58.7 ± 2.5	0.06 ± 0.05	-34.4	-24.3
	WT	5.2 ± 1.2	-45.4 ± 6.3	0.01 ± 0.12	-24.2	-21.2
	Abl-SH3	323	-91.6	0.1	-60.2	-31.4

3.0	R21A Spc-SH3	18.2 ± 2.0	-51.3 ± 1.7	-0.29 ± 0.08	-27.0	-24.3
	WT Spc-SH3	5.8 ± 0.8	-43.9 ± 3.3	-0.07 ± 0.11	-22.4	-21.5
	Abl-SH3	210	-93.3	-	-62.9	-30.4

^1^The similar parameters for the Abl-SH3 domain are also shown for comparison and are taken from reference 16. The errors in each parameter were estimated from 95% confidence intervals as determined from the fittings of the binding isotherms.

**Table 2:** Structural statistics for the 20 conformers of R21A Spc-SH3 in its free and p41-bound form^1^

	SH3 free form	SH3 bound form
**Distance restraints^2^**		

Intraresidue (i-j = 0)	630	591
Sequential (|i-j| = 1)	338	337
Medium range (2 ≤ |i-j| = ≤ 4)	133	114
Long range (|i-j| ≥ 5)	573	465
Hydrogen bonds	2 × 23	2 × 22
All	1720	1551

**Restraint statistics**		

NOE RMSD (10^-2 ^Å)	2.93 +/- 0.26	3.15 +/- 0.48
violations > 0.5 Å	0	0.25 +/- 0.43
violations > 0.3 Å	1.60 +/- 1.28	1.55 +/- 1.43
violations > 0.1 Å	37.8 +/- 6.5	34.5 +/- 6.9

**RMSD from idealized covalent geometry**		

Bonds (Å)	0.0045 +/- 0.00016	0.0043 +/- 0.00018
Angles (°)	0.596 +/- 0.016	0.587 +/- 0.022
Impropers (°)	1.53 +/- 0.10	1.44 +/- 0.13
Dihedrals (°)	40.6 +/- 0.2	40.5 +/- 0.2

**CNS energies (kcal/mol)**		

E_total_	-2507.4 +/- 57.8	-2511.7 +/- 66.3
E_vdw_	-572.8 +/- 6.1	-569.0 +/- 12.8
E_elec_	-2567.1 +/- 56.6	-2545.5 +/- 67.0

**Pairwise RMSD^3 ^of residues 7–61 in Å**		

Backbone N,CA,C'	0.34 +/- 0.06	0.41 +/- 0.08
Heavy atoms	0.78 +/- 0.09	0.97 +/- 0.14

**Ramachandran plot**^4^		

Most favored regions (%)	86.9	88.6
Additional allowed regions (%)	8.5	9.2
Generously allowed regions (%)	0.2	0.4
Disallowed regions (%)	4.4	1.8

^1^The statistics is obtained from an ensemble of twenty lowest-energy water-refined solution structures for the free and bound form of R21A Spc-SH3.

^2^Distance restraint statistics reported for unique, unambiguous assigned NOEs.

^3^Coordinate precision is given as the average pair-wise Cartesian coordinate Root Mean Square Deviations over the ensemble.

^4^Values obtained from the PROCHECK analysis^46 ^excluding the first six highly flexible residues.

**Table 3:** Structural statistics for the 20 conformers of the R21A Spc-SH3:P41 complex^1^

**Distance restraints**	
Ambiguous Interaction Restraints	33
Intra-molecular NOE-derived Restraints:	
SH3	1507
p41	17
Intermolecular NOE-derived Restraints	38
All	1595

**Violations**^2^	

violations > 0.3 Å	0
violations > 0.1 Å	0.2 +/- 0.4

**RMSD from idealized covalent geometry**	

Bonds (Å)	0.0051 +/- 0.00026
Angles (°)	0.617 +/- 0.025
Impropers (°)	0.531 +/- 0.019
Dihedrals (°)	21.6 +/- 0.9

**CNS Interaction energies (kcal/mol)**	

E_int_	-92.98 +/- 3.06
E_vdw_	- 47.02 +/- 2.09
E_elec_	-45.96 +/- 3.95
Buried Surface Area (Å^2^)	940.6 +/- 15.3

**Pairwise r.m.s.d.^3 ^in Å**	

Backbone N,CA,C' of residues 7–61 of SH3	0.24 +/- 0.05
Heavy atoms of residues 7–61 of SH3	0.63 +/- 0.17
Interface backbone N,CA,C'	0.460

**Ramachandran plot**^4^	

Most favored regions (%)	86.2
Additional allowed regions (%)	11.4
Generously allowed regions (%)	1.2
Disallowed regions (%)	1.3

^1^The statistics is obtained from an ensemble of twenty water-refined solution structures for the R21A Spc-SH3:P41 complex.

^2^Violations reported only for the interaction, p41 intra-molecular and intermolecular NOE-derived restraints.

^3^Coordinate precision is given as the average pairwise Cartesian coordinate root mean square deviations over the ensemble. The interface is defined as residues 7–61 of R21A Spc-SH3 and residues 1–10 of p41. These sequence regions were also used for clustering.

^4^Values obtained from the PROCHECK analysis^46 ^for the SH3 domain excluding the first six highly flexible residues.

**Table 4:** Intermolecular contacts^1 ^statistics calculated over the ensemble of twenty R21A Spc-SH3:P41 complex structures.

**Interacting residues**		**Hydrogen bonds**			**Non-bonded contacts**	**Occurrence Total**	**Interacting residue in Abl-SH3**
**p41**	**Spc-SH3**	**M-M**	**S-S**	**M-S**		**%**	
Ace0	Phe52	0	0	0	2	70	Trp47 (2, 75%)
Ala1	Phe52	0	0	0	3	95	Trp47 (6, 100%)
Pro2	Trp41	0	0	0	7	100	Trp36 (2, 100%)
Pro2	Phe52	0	0	0	2	50	Trp47 (2, 50%)
Tyr4	Lys18	0	1	0	0	100	Ser12 (0, 75%)
Tyr4	Trp41	0	0	0	1	95	Trp36 (1, 75%)
Tyr4	Trp41	0	0	1	0	100	Trp36 (0, 100%)
Pro6	Pro54	0	0	0	1	70	Pro49 (2, 100%)
Pro6	Asp40	0	0	0	2	100	Glu35 (2, 50%)
Pro6	Trp41	0	0	0	3	100	Trp36 (5, 100%)
Pro7	Tyr15	0	0	0	1	80	Phe9 (2, 100%)
Pro8	Tyr57	0	0	1	0	15	Tyr52 (0, 100%)
Pro9	Tyr13	0	0	0	2	100	Tyr7 (2, 100%)
Pro9	Tyr57	0	0	0	3	100	Tyr52 (1, 100%)
Pro10	Tyr13	0	0	0	2	65	Tyr7 (7, 100%)
Pro10	Tyr57	0	0	0	1	40	Tyr52 (1, 75%)
Pro7	Tyr57	0	0	1	0	95	-
Ala1	-	0	0	0	-	-	Asn31 (1, 100%)
Ala1	-	0	0	0	-	-	Trp36 (1, 75%)
Pro2	-	0	0	0	-	-	Thr16 (1, 100%)
Tyr4	-	0	1	0	-	-	Asp14 (1, 75%)
Ser5	-	0	0	0	-	-	Trp36 (1, 50%)

^1^Intermolecular contacts were analyzed with LIGPLOT [24] using the default settings: 2.7Å and 3.35Å proton-acceptor and donor-acceptor distance cut-offs, respectively with minimum 90° angles for hydrogen bonds; 3.9Å heavy atom pair distance cut-off for non-bonded contacts. Contacts are reported if present in at least 10 of the 20 structures or in at least 1 of the 20 structures when the corresponding contact in Abl-SH3 is present in 2 or more of the 4 models in the pdb entry 1BBZ. Hydrogen bonds are reported for main-chain to main-chain (M-M), side-chain to side-chain (S-S) and main-chain to side-chain (M-S). The average number of non-bonded contacts is reported, as well as the percentage of occurrences of a contact. The corresponding interacting residue in Abl-SH3 for a certain contact and the number of contacts and occurrence in the 4 models (between brackets, respectively) are given in the last column. The last five rows correspond to contacts present in Abl-SH3:P41 that are not present in any of the 20 structures reported here.

**Table 5:** Structure-based calculation of the thermodynamic parameters of binding between p41 and R21A Spc-SH3 and Abl-SH3 domain.

**Protein**	**Buried water molecules^1^**	**ΔH_b _(kJ mol^-1^)**	**T ΔS_b _(kJ mol^-1^)**	**ΔG_b _(kJ mol^-1^)**	**ΔC_p,b _(kJ K^-1^mol^-1^)**
R21A Spc-SH3	No water	28.5	46.5	-18.0	-1.0
	2 bound water molecules	-1.0	27.4	-28.4	-0.84

Abl-SH3	No water	21.6	41.4	-19.8	-0.98
	5 bound water molecules	-58.5	-9.6	-49.1	-0.47

^1^Water molecules present in the bound state and not present in the unbound state were considered to contribute to the solvent accessible surface area of the unbound state as a fully exposed molecule.
