# Supplementary material for: The high-resolution NMR structure of the R21A Spc-SH3:P41 complex: Understanding the determinants of binding affinity by comparison with Abl-SH3
Source: BMC Struct Biol. 2007 Apr 2;7:22. doi: 10.1186/1472-6807-7-22 (PMC1853097; doi:10.1186/1472-6807-7-22)
Supplement: Additional file 1 — Thermodynamic analysis of protein-ligand binding using differential scanning calorimetry and two tables: Table 1: Ambiguous interaction restraints (AIR) and intermolecular NOE-derived distance restraints. Table 2: Apparent amide hydrogen-deuterium exchange rate constants and apparent Gibbs energies for the R21A Spc-SH3 domain at pH* 3.0 and 27.1°C, in its free form and in the presence of a 96% saturating concentration of the p41 peptide. description and equations used for analysis of DSC thermograms and list of AIR and SH3:P41 intermolecular NOEs and a list of amide hydrogen-deuterium exchange rate constants and apparent Gibbs energies. [file 1472-6807-7-22-S1.doc]

**Thermodynamic analysis of protein-ligand binding using differential scanning calorimetry**

Here we present the detailed derivation of the equations used to analyze the differential scanning calorimetry curves for different protein/ligand molar ratios. We have used a simple model with two coupled equilibria, i.e., the protein-ligand binding/dissociation equilibrium and the two-state folding/unfolding of the protein:

$$NL \xleftrightarrow{} N+L \xleftrightarrow{} U+L$$

In this scheme N is the native free protein, U is the unfolded protein, L is the free ligand and NL is the protein-ligand complex. The relevant binding and unfolding equilibrium constants are defined as:

$$K_{b}=\frac{\left[NL\right]}{\left[N\right]\cdot\left[L\right]}$$

$$K_{u}=\frac{\left[U\right]}{\left[N\right]}$$

and using the native state, N, as the reference state of the protein subsystem, the partition function of the protein and its temperature derivative are given by:

$$Q=\frac{\left[N\right]+\left[U\right]+\left[NL\right]}{\left[N\right]}=1+K_{u}+K_{b}\left[L\right]$$

$$\frac{dQ}{dT}=K_{u}\frac{\Delta H_{u}}{RT^{2}}+K_{b}\left[L\right]\frac{\Delta H_{b}}{RT^{2}}+K_{b}\frac{d\left[L\right]}{dT}$$

The molar fractions of protein in each state can be obtained as:

$$x_{N}=\frac{1}{Q}$$

$$x_{U}=\frac{K_{u}}{Q}$$

$$x_{NL}=\frac{K_{b}\left[L\right]}{Q}$$

and the total concentration of ligand, L_0_, is:

$$L_{0}=\left[L\right]+\left[NL\right]=\left[L\right]\cdot\left(1+\frac{K_{b}C_{0}}{Q}\right)$$

where C_0_ is the total protein concentration in the solution. Substituting Q and solving for the free ligand concentration, [L]:

$$\left[L\right]=\frac{-B+\sqrt{B^{2}-4AC}}{2A}$$

where A, B and C are, respectively:

$$A=K_{b}$$

$$B=K_{b}\left(C_{0}-L_{0}\right)+K_{u}+1$$

$$C=-L_{0}\left(1+K_{u}\right)$$

The temperature derivatives of each of these quantities are:

$$\frac{dA}{dT}=\frac{dK_{b}}{dT}=K_{b}\frac{\Delta H_{b}}{RT^{2}}$$

$$\frac{dB}{dT}=\frac{dK_{b}}{dT}\left(C_{0}-L_{0}\right)+\frac{dK_{u}}{dT}=K_{b}\frac{\Delta H_{b}}{RT^{2}}\left(C_{0}-L_{0}\right)+K_{u}\frac{\Delta H_{u}}{RT^{2}}$$

$$\frac{dC}{dT}=-L_{0}\frac{dK_{u}}{dT}=-L_{0}K_{u}\frac{\Delta H_{u}}{RT^{2}}$$

and the temperature derivative of [L] is then given by:

$$\begin{matrix} \frac{d\left[L\right]}{dT}=\frac{\left[-\frac{dB}{dT}+\left(B^{2}-4AC\right)^{-\frac{1}{2}}\left(B\frac{dB}{dT}-2C\frac{dA}{dT}-2A\frac{dC}{dT}\right)\right]}{2A} \\ -\frac{2\frac{dA}{dT}\left[-B+\left(B^{2}-4AC\right)^{\frac{1}{2}}\right]}{4A^{2}} \end{matrix}$$

We define the average enthalpy of the whole system as:

$$\left\langle H\right\rangle=H_{N}\left[N\right]+H_{NL}\left[NL\right]+H_{U}\left[U\right]+H_{L}\left[L\right]$$

where H_N_, H_NL_, H_U_ and H_L_ and the molar enthalpies of each species in the solution. If we use as the reference state for the whole system a hypothetical state where all the protein is in its native and free state and all the ligand is also free, the enthalpy of this reference state would be:

$$\left\langle H\right\rangle_{ref}=H_{N}C_{0}+H_{L}L_{0}$$

and the excess enthalpy relative to this reference state is:

$$\begin{matrix} \left\langle\Delta H\right\rangle=H_{N}\left(\left[N\right]-C_{0}\right)+H_{U}\left[U\right]+H_{NL}\left[NL\right]+H_{L}\left(\left[L\right]-L_{0}\right) \\ =\left(H_{U}-H_{N}\right)\left[U\right]+\left(H_{NL}-H_{N}-H_{L}\right)\left[NL\right]=\Delta H_{u}\left[U\right]+\Delta H_{b}\left[NL\right] \end{matrix}$$

Dividing by the total protein concentration, C_0_:

$$\left\langle\Delta H\right\rangle=\Delta H_{u}x_{U}+\Delta H_{b}\cdot x_{NL}$$

which is expressed per mole of protein.

The excess heat capacity, ΔC_p_, is the temperature derivative of the excess enthalpy:

$$\Delta C_{p}=\frac{d\left\langle H\right\rangle}{dT}=\Delta C_{p,u}\cdot x_{U}+\Delta H_{u}\frac{dx_{U}}{dT}+\Delta C_{p,b}\cdot x_{nL}+\Delta H_{b}\frac{dx_{NL}}{dT}$$

where the temperature derivatives of the mole fractions are given by:

$$\frac{dx_{U}}{dT}=\frac{K_{u}}{Q}\frac{\Delta H_{u}}{RT^{2}}-\frac{dQ}{dT}\cdot\frac{K_{u}}{Q^{2}}$$

$$\frac{dx_{NL}}{dT}=\frac{1}{Q}\left(K_{b}\frac{\Delta H_{b}}{RT^{2}}\left[L\right]+K_{b}\frac{d\left[L\right]}{dT}\right)-\frac{K_{b}\left[L\right]}{Q^{2}}\frac{dQ}{dT}$$

It is necessary to define the molar heat capacity functions for each state of the system.

We have assumed linear functions for the native state of the protein and the protein-ligand complex and a quadratic function for the unfolded protein. This last function can be calculated from the protein sequence using the parametrization of Makhatadze and Privalov (Makhatadze, G. I. & Privalov, P. L. (1990) J. Mol. Biol. 213, 375-384). For the free ligand, we determined experimentally its C_p_ function, which is accurately described by a 4^th^ order polynomial.

$$C_{p}(N)=n0+n1\cdot T$$

$$C_{p}(NL)=nl0+nl1\cdot T$$

$$C_{p}(U)=u0+u1\cdot T+u2\cdot T^{2}$$

$$C_{p}(L)=l0+l1\cdot T+l2\cdot T^{2}+l3\cdot T^{3}+l4\cdot T^{4}$$

Accordingly, the temperature functions for the heat capacity changes of unfolding and binding are:

$\Delta C_{p,u}=(u0-n0)+(u1-n1)\cdot T+u2\cdot T^{2}$

$\Delta C_{p,b}=(nl0-n0-l0)+(nl1-n1-l1)\cdot T-l2\cdot T^{2}-l3\cdot T^{3}-l4\cdot T^{4}$

and the temperature dependences of the enthalpy, entropy and Gibbs energy changes as well as of the equilibrium constant for the unfolding process are given by:

$$\Delta H_{u}=\Delta H_{u}(T_{u})+(u0-n0)\cdot(T-T_{u})+\frac{(u1-n1)}{2}\cdot(T^{2}-T_{u}^{2})+\frac{u2}{3}(T^{3}-T_{u}^{3})$$

$$\Delta S_{u}=\frac{\Delta H_{u}(T_{u})}{T_{u}}+(u0-n0)\ln\frac{T}{T_{u}}+(u1-n1)\cdot(T-T_{u})+\frac{u2}{2}(T^{2}-T_{u}^{2})$$

$$\Delta G_{u}=\Delta H_{u}-T\cdot\Delta S_{u}$$

$$K_{u}=e^{-\frac{\Delta G_{u}}{RT}}$$

where T_u_ is the unfolding temperature of the free protein, i.e., K_u_(T_u_) = 1.

Similarly, the temperature dependences of the enthalpy change and the equilibrium constant of binding are given by:

$$\begin{matrix} \Delta H_{b}=\Delta H_{b}(T_{b})+(nl0-n0-l0)\cdot(T-T_{b})+(nl1-n1-l1)\cdot\frac{(T^{2}-T_{b}^{2})}{2}-\frac{l2}{3}(T^{3}-T_{b}^{3}) \\ -\frac{l3}{4}(T^{4}-T_{b}^{4})-\frac{l4}{5}(T^{5}-T_{b}^{5}) \end{matrix}$$

$\begin{matrix} \ln K_{b}=\ln K_{b}(T_{b}) \\ -\frac{\Delta H_{b}(T_{b})}{R}+\frac{(nl0-n0-l0)}{R}\cdot T_{b}+\frac{(nl1-n1-l1)}{2R}\cdot T_{b}^{2}-\frac{l2}{3R}\cdot T_{b}^{3}-\frac{l3}{4R}\cdot T_{b}^{4}-\frac{l4}{5R}\cdot T_{b}^{5}\left(\frac{1}{T}-\frac{1}{T_{b}}\right) \\ +\frac{(nl0-n0-l0)}{R}\ln\frac{T}{T_{b}}+\frac{(nl1-n1-l1)}{2R}(T-T_{b})-\frac{l2}{6R}(T^{2}-T_{b}^{2})-\frac{l3}{12R}(T^{3}-T_{b}^{3}) \\ -\frac{l4}{20R}(T^{4}-T_{b}^{4}) \end{matrix}$

where T_b_ is reference temperature where K_b_(T_b_) and ΔH_b_ are known.

Finally, the molar partial heat capacity of the whole system, C_p_, expressed per mole of protein, is:

$$C_{p}=C_{p}(N)+C_{p}(L)\frac{L_{0}}{C_{0}}+\Delta C_{p}$$

from which the apparent heat capacity curve measured in a DSC experiment relative to the baseline obtained for the buffer, $\Delta C_{p}^{app}$, can be derived as:

$$\begin{matrix} \Delta C_{p}^{app}=C_{p}-\frac{\text{v}_{M}}{\text{v}_{H_{2}O}}C_{p}(H_{2}O)-\frac{\text{v}_{L}}{\text{v}_{H_{2}O}}C_{p}(H_{2}O)\frac{L_{0}}{C_{0}}= \\ C_{p}(N)-\frac{\text{v}_{M}}{\text{v}_{H_{2}O}}C_{p}(H_{2}O)+\left(C_{p}(L)-\frac{\text{v}_{L}}{\text{v}_{H_{2}O}}C_{p}(H_{2}O)\right)\frac{L_{0}}{C_{0}}+\Delta C_{p} \end{matrix}$$

We have considered the partial specific volumes of the ligand and the protein equal to 0.73 ml g^−1^.

| **Table 1: Ambiguous interaction and intermolecular NOE-derived distance restraints** |
| --- |
| AIRs of protons of SH3 to all atoms of ligand within 6 Å Leu12.HA, Leu12.HB  Tyr13.HA, Tyr13.HB  Tyr15.HD  Gln16.NH  Lys18.NH  Ala21.NH  Glu22.NH  Asn38.HB, Asn38.HD2  Asp40.NH, Asp40.HB  Trp41.NH, Trp41.HB, Trp41.HE3, Trp41.HE1  Trp42.NH  Lys43.HA, Lys43.HB  Phe52.NH, Phe52.HA, Phe52.HB, Phe52.HE  Pro54.HA, Pro54.HB, Pro54.HD  Ala55.NH  Ala56.NH, Ala56.HB  Tyr57.NH, Tyr57.HB, Tyr57.HD |
| Intermolecular NOEs: R21A-SH3 - P41 ligand Tyr15.HE# - Pro7.HD#  Asn38.HD21 - Ala1.HB#  Asn38.HD22 - Ala1.HB#  Asp40.HB1 - Pro6.HD1  Asp40.HB1 - Pro6.HD2  Trp41.HE3 - Ala1.HA  Trp41.HD1 - Ala1.HB#  Trp41.HE1 – Ser3.HA  Trp41.HE1 - Tyr4.HD#  Trp41.HH2 - Tyr4.HE#  Trp41.HZ2 - Tyr4.HD#  Trp41.HZ2 - Tyr4.HE#  Trp41.HD1 - Ser5.HA  Trp41.HE1 - Ser5.HA  Trp41.HZ2 - Ser5.HA  Trp41.HE1 - Pro6.HA  Trp41.HH2 - Pro6.HA  Trp41.HZ2 - Pro6.HA  Trp41.HD1 - Pro6.HD1  Trp41.HD1 - Pro6.HD2  Trp41.HE1 - Pro6.HD1  Trp41.HE1 - Pro6.HD2  Trp41.HZ2 - Pro6.HD1  Trp41.HZ2 - Pro6.HD2  Trp41.HH2 - Pro7.HD#  Trp41.HZ2 - Pro7.HD#  Phe52.HD# - Ace0.HA#  Phe52.HE# - Ace0.HA#  Phe52.HZ – Ace0.HA#  Phe52.HD# - Ala1.HB#  Phe52.HE# - Ala1.HA  Phe52.HE# - Ala1.HB#  Phe52.HE# - Ala1.HN  Phe52.HZ - Ala1.HN  Phe52.HZ - Ala1.HA  Phe52.HZ - Ala1.HB#  Tyr57.HD# - Pro9.HD#  Tyr57.HE# - Pro9.HD# |

| **Table 2.** Apparent amide hydrogen-deuterium exchange rate constants and apparent Gibbs energies for the R21A Spc-SH3 domain at pH* 3.0 and 27.1 ºC, in its free form and in the presence of a 96% saturating concentration of the p41 peptide. Uncertainties in the values correspond to 95% confidence intervals for the k_hx_ values. | | | | |
| --- | --- | --- | --- | --- |
|  | Free R21A Spc-SH3 | | R21A Spc-SH3 + p41 | |
| Residue | k_hx_ · 10^-3^  (min^-1^) | ΔG_hx_  (kJ·mol^−1^) | k_hx_ · 10^-3^  (min^-1^) | ΔG_hx_  (kJ·mol^−1^) |
| Leu 8 | 8.5 ± 0.6 | 5.29 ± 0.19 | 5.2 ± 0.3 | 6.50 ± 0.15 |
| Val 9 | 1.13 ± 0.05 | 6.81 ± 0.12 | 0.044 ± 0.004 | 14.73 ± 0.21 |
| Leu 10 | 1.18 ± 0.07 | 7.58 ± 0.14 | 0.035 ± 0.008 | 16.2 ± 0.6 |
| Ala 11 | 2.2 ± 0.3 | 9.1 ± 0.3 | 0.01 ± 0.03 | 16.8 ± 0.9 |
| Leu 12 | 1.34 ± 0.11 | 8.22 ± 0.21 | 0.031 ± 0.011 | 17.4 ± 0.8 |
| Tyr 13 | 1.42 ± 0.05 | 8.56 ± 0.09 | 0.042 ± 0.005 | 17.2 ± 0.3 |
| Asp 14 | - | - | 5.1 ± 0.6 | 10.9 ± 0.3 |
| Tyr 15 | 3.2 ± 0.5 | 10.1 ± 0.4 | 0.097 ± 0.023 | 18.6 ± 0.6 |
| Gln 16 | 20.3 ± 1.0 | 4.98 ± 0.12 | 4.41 ± 0.16 | 8.74 ± 0.09 |
| Glu 17 | 23.9 ± 0.5 | 6 ± 3 | - | - |
| Ser 19 | 35 ± 14 | 5.7 ± 1.1 | 29 ± 5 | 6.2 ± 0.5 |
| Glu 22 | 28 ± 2 | 4.62 ± 0.24 | 6.8 ± 0.4 | 8.15 ± 0.13 |
| Val 23 | 5.05 ± 0.16 | 5.87 ± 0.08 | 1.00 ± 0.03 | 9.85 ± 0.09 |
| Thr 24 | 14.4 ± 0.8 | 4.02 ± 0.14 | 4.42 ± 0.17 | 6.95 ± 0.10 |
| Met 25 | 8.7 ± 0.3 | 7.46 ± 0.10 | 0.33 ± 0.03 | 15.51 ± 0.20 |
| Lys 26 | 9.3 ± 0.6 | 6.67 ± 0.17 | 2.54 ± 0.08 | 9.87 ± 0.08 |
| Gly 28 | 9.9 ± 1.8 | 8.3 ± 0.5 | 1.80 ± 0.18 | 12.52 ± 0.24 |
| Asp29 | 8 ± 3 | 10.5 ± 1.0 | - | - |
| Ile 30 | 5.9 ± 0.3 | 5.95 ± 0.11 | 1.69 ± 0.08 | 9.01 ± 0.12 |
| Leu 31 | 1.26 ± 0.06 | 6.78 ± 0.12 | 0.033 ± 0.005 | 15.7 ± 0.4 |
| Thr 32 | 2.9 ± 0.3 | 7.6 ± 0.3 | 0.137 ± 0.011 | 15.27 ± 0.22 |
| Leu 33 | 2.06 ± 0.19 | 7.86 ± 0.23 | 0.072 ± 0.008 | 16.1 ± 0.3 |
| Leu 34 | 1.5 ± 0.3 | 6.8 ± 0.4 | 0.059 ± 0.009 | 14.8 ± 0.4 |
| Asn 35 | 9 ± 3 | 7.9 ± 0.7 | 0.58 ± 0.07 | 14.7 ± 0.3 |
| Thr 37 | 49 ± 12 | 3.4 ± 0.6 | 9.3 ± 0.6 | 7.55 ± 0.17 |
| Asn 38 | 26 ± 5 | 7.6 ± 0.5 | 5.5 ± 0.4 | 11.4 ± 0.17 |
| Asp40 | 3.1 ± 1.9 | 12.5 ± 1.5 | - | - |
| Trp 41 | 5.83 ± 0.19 | 7.85 ± 0.08 | 0.269 ± 0.021 | 15.4 ± 0.19 |
| Trp 42 | 0.69 ± 0.05 | 9.90 ± 0.19 | 0.026 ± 0.009 | 17.9 ± 0.8 |
| Lys 43 | 1.85 ± 0.07 | 9.38 ± 0.10 | 0.047 ± 0.007 | 18.4 ± 0.4 |
| Val 44 | 1.08 ± 0.04 | 8.40 ± 0.10 | 0.030 ± 0.007 | 17.2 ± 0.4 |
| Glu 45 | 4.08 ± 0.19 | 8.54 ± 0.12 | 0.102 ± 0.007 | 17.58 ± 0.18 |
| Val 46 | 2.05 ± 0.10 | 8.07 ± 0.12 | 0.079 ± 0.004 | 16.04 ± 0.14 |
| Arg 49 | 10.8 ± 0.4 | 8.93 ± 0.09 | 2.05 ± 0.05 | 13.01 ± 0.06 |
| Gln 50 | 11.36 ± 0.3 | 7.33 ± 0.06 | 3.63 ± 0.09 | 10.16 ± 0.06 |
| Gly 51 | 8.6 ± 1.2 | 9.1 ± 0.4 | 0.22 ± 0.04 | 18.1 ± 0.4 |
| Phe 52 | 6.61 ± 0.4 | 6.97 ± 0.15 | 0.31 ± 0.05 | 14.4 ± 0.4 |
| Val 53 | 1.42 ± 0.21 | 7.4 ± 0.4 | 0.034 ± 0.007 | 16.5 ± 0.5 |
| Ala 55 | 3.1 ± 0.6 | 8.1 ± 0.5 | 0.13 ± 0.03 | 15.9 ± 0.6 |
| Ala 56 | 10.8 ± 1.8 | 6.4 ± 0.4 | 3.8 ± 0.3 | 8.94 ± 0.18 |
| Tyr 57 | 1.83 ± 0.12 | 9.05 ± 0.17 | 0.047 ± 0.009 | 18.0 ± 0.5 |
| Val 58 | 0.96 ± 0.03 | 8.29 ± 0.07 | 0.020 ± 0.007 | 17.8 ± 0.8 |
| Lys 59 | 2.81 ± 0.11 | 8.23 ± 0.10 | 0.074 ± 0.007 | 17.1 ± 0.2 |
| Lys 60 | 26 ± 8 | 4.2 ± 0.8 | 11 ± 3 | 6.3 ± 0.6 |
| Leu 61 | 8.3 ± 0.4 | 4.12 ± 0.12 | 5.3 ± 0.2 | 5.24 ± 0.10 |
